# Calcium Channel Blocker Enhances Beneficial Effects of an Angiotensin II AT_1_ Receptor Blocker against Cerebrovascular-Renal Injury in type 2 Diabetic Mice

**DOI:** 10.1371/journal.pone.0082082

**Published:** 2013-12-10

**Authors:** Kazi Rafiq, Shamshad J. Sherajee, Hirofumi Hitomi, Daisuke Nakano, Hiroyuki Kobori, Koji Ohmori, Hirohito Mori, Hideki Kobara, Tsutomu Masaki, Masakazu Kohno, Akira Nishiyama

**Affiliations:** 1 Department of Pharmacology, Faculty of Medicine, Kagawa University, Kagawa, Japan; 2 Cardiorenal and Cerebrovascular Medicine, Faculty of Medicine, Kagawa University, Kagawa, Japan; 3 Gastroenterology, Faculty of Medicine, Kagawa University, Kagawa, Japan; Biological Research Centre of the Hungarian Academy of Sciences, Hungary

## Abstract

Recent clinical trials have demonstrated that combination therapy with renin-angiotensin system inhibitors plus calcium channel blockers (CCBs) elicits beneficial effects on cardiovascular and renal events in hypertensive patients with high cardiovascular risks. In the present study, we hypothesized that CCB enhances the protective effects of an angiotensin II type 1 receptor blocker (ARB) against diabetic cerebrovascular-renal injury. Saline-drinking type 2 diabetic KK-A^y^ mice developed hypertension and exhibited impaired cognitive function, blood-brain barrier (BBB) disruption, albuminuria, glomerular sclerosis and podocyte injury. These brain and renal injuries were associated with increased gene expression of NADPH oxidase components, NADPH oxidase activity and oxidative stress in brain and kidney tissues as well as systemic oxidative stress. Treatment with the ARB, olmesartan (10 mg/kg/day) reduced blood pressure in saline-drinking KK-A^y^ mice and attenuated cognitive decline, BBB disruption, glomerular injury and albuminuria, which were associated with a reduction of NADPH oxidase activity and oxidative stress in brain and kidney tissues as well as systemic oxidative stress. Furthermore, a suppressive dose of azelnidipine (3 mg/kg/day) exaggerated these beneficial effects of olmesartan. These data support the hypothesis that a CCB enhances ARB-associated cerebrovascular-renal protective effects through suppression of NADPH oxidase-dependent oxidative stress in type 2 diabetes.

## Introduction

The beneficial effects of renin-angiotensin system (RAS) inhibition with angiotensin converting enzyme inhibitors (ACEIs) and angiotensin II type 1 receptor blockers (ARBs) have been demonstrated in hypertensive patients with high cardiovascular-renal risks including heart failure [1], myocardial infarction [2], diabetes [3] and chronic kidney disease (CKD) [4]. Accordingly, most national guideline groups have recommended the use of RAS inhibitors in preference to other antihypertensive agents for high-risk hypertensive patients [5-9]. However, treatment with multiple antihypertensive medications is often necessary to attain recommended blood pressure control [10], and additional administration of other antihypertensive drugs, including calcium channel blockers (CCBs) and diuretics, or treatment with high-dose RAS inhibitors has been suggested in high-risk hypertensive patients who are treated with RAS inhibitors [11]. Recently, the ACCOMPLISH study showed that concomitant use of RAS inhibitors and CCBs more effectively reduced cardiovascular events than did RAS inhibitors and diuretics in high-risk hypertensive patients [12]. Similarly, the combination of an ARB and CCB reduces the incidence of composite cardiovascular events compared with a high-dose of an ARB in hypertensive patients with cardiovascular disease [13] and CKD [4].

Previous large-scale epidemiological studies have indicated that CKD is not related to the incidence of stroke [14]. However, a growing body of evidence has indicated the potential relationship between CKD and cerebrovascular injury; this is known as the cerebrovascular-renal connection [15-17]. Wada et al. [18] performed brain magnetic resonance imaging in community-based elderly subjects and showed that the levels of albuminuria were highly correlated with the degree of cerebral small vessel disease but independent of other risk factors such as hypertension and diabetes. Similarly, silent brain infarction is an independent prognostic factor for the progression of renal injury in patients with CKD [19]. Further studies have shown that in CKD patients with first-ever ischemic stroke, proteinuria independently contributes to the increased risk of neurologic deterioration [20]. Histological evaluations in salt-treated hypertensive rats have also revealed that cerebral small vessel injury is associated with juxtamedullary glomerular podocyte injury and albuminuria during the development of hypertension [17].

It has been highlighted that type 2 diabetes and hypertension are risk factors for cerebrorenal injury including cognitive decline [21,22], BBB disruption [23] and nephropathy [24]. Therefore, the present study was conducted to investigate whether cognitive impairment, BBB disruption and renal injury occur simultaneously in type 2 diabetic mice. Studies were also performed to test the hypothesis that CCBs enhance the protective effects of an ARB against cerebrovascular-renal injury. We examined effects of suppressive doses of the CCB, azelnidipine [25], on cognitive impairment, BBB disruption and renal injury in salt-treated type 2 diabetic KK-A^y^ mice treated with the ARB, olmesartan [26].

## Materials and Methods

### Animals

Experimental protocols and animal care were performed according to the guidelines for the care and use of animals established by Kagawa University, Japan. The experiments were approved by the Animal Experimentation Ethics Committee at Kagawa University (No. 112). At the end of the experiment, organs were dissected under sodium pentobarbital anesthesia (65 mg/kg, i.p.). Six-week-old male type 2 diabetic KK-A^y^ mice [27] and control C57BL/6J mice (CLEA Japan Inc., Tokyo, Japan) were used in this study. Mice were maintained in a specific pathogen-free facility under a controlled temperature (24±2°C) and humidity (55±5%) with a 12-hour light/dark cycle. 

### Experimental protocols

After a two week period of acclimatization and measuring basal parameters, 8-week-old mice were underwent combination treatments for 16 weeks. Control C57BL6 mice were divided into two groups: tap water drinking C57BL6 mice (C57BL6, *n* = 11) and saline-drinking C57BL6 mice (C57BL6 + 0.9% NaCl, *n* =11). Type 2 diabetic KK-A^y^ mice were divided into four groups: tap water drinking KK-A^y^ mice (KK-A^y^, *n* = 11); saline-drinking KK-A^y^ mice (KK-A^y^ + 0.9% NaCl, *n* = 11); saline-drinking KK-A^y^ mice treated with olmesartan (0.009% in laboratory chow, approximately 10 mg/kg body weight/day; Daiichi-Sankyo Co., Ltd., Tokyo, Japan) (KK-A^y^ + 0.9% NaCl + olmesartan, *n* = 11); saline-drinking KK-A^y^ mice treated with olmesartan plus azelnidipine (0.0027% in laboratory chow, approximately 3 mg/kg body weight/day; Daiichi-Sankyo Co., Ltd., Tokyo, Japan) (KK-A^y^ + 0.9% NaCl + olmesartan + azelnidipine, *n* = 11). It has previously been reported that azelnidipine at 3 mg/kg body weight/day did not change blood pressure in KK-A^y^ mice [25]. Furthermore, our preliminary studies showed similar blood pressure reduction in response to olmesartan (10 mg/kg/day) alone, olmesartan + azelnidipine (3 mg/kg/day) or hydralazine (25 mg/kg/day) in saline-drinking KK-A^y^ mice (*n* = 5 for each, data not shown).

Systolic blood pressure (SBP) was monitored in conscious mice by tail-cuff plethysmography (BP-98A; Softron Co., Tokyo, Japan). Urinary albumin and creatinine concentrations were measured by using commercially available assay kits (SHIBAYAGI Co., Ltd., Shibukawa, Japan, and micro CRE-test; Wako Pure Chemical Industries, Ltd., Osaka, Japan, respectively) [26]. Postprandial blood glucose (PPBG) was measured with a glucometer (Sanwa-Kagaku Co., Ltd., Nagoya, Japan).

### Sample collection

At the end of the experiment blood, brain and kidney samples were harvested under anesthesia with sodium pentobarbital (65 mg/kg, i.p.). The brain and kidney tissues were harvested and fixed in 10% buffered paraformaldehyde or embedded in Tissue-Tek OCT compound (Sakura Finetek Japan Co., Ltd., Tokyo, Japan), and remaining tissues were snap-frozen in liquid nitrogen. Small amounts of brain and renal cortical tissues were collected in RNAlater and stored overnight at 4°C RNAlater-treated samples were subsequently snap-frozen in liquid nitrogen and stored at -80°C until processing for RNA extraction and reverse transcription-polymerase chain reaction (RT-PCR) analysis.

### Passive avoidance test

A passive avoidance test was performed to evaluate cognitive function as described previously [28]. Details are provided in the supplemental methods (Methods S1).

### Evans Blue (EB) assay

BBB permeability was determined using the EB extravasation technique (*n* = 6 per group) as previously described [29,30]. Details are provided in the supplemental methods (Methods S1).

### Histopathologic examination

Kidney tissues were fixed with 10% paraformaldehyde, embedded in paraffin, sectioned into 4-µm-thick slices, and stained with periodic acid-Schiff (PAS) and Mallory-Azan reagents to evaluate glomerular sclerosis and tubulointerstitial fibrosis, respectively [31,32]. The percentage of PAS-positive areas was measured in each experimental group using image analysis software, WinROOF (Mitani Corp., Ltd., Tokyo, Japan). A total of 20–25 glomeruli were examined per mouse and the average percentage of affected lesions were calculated [31,32]. The extent of the interstitial fibrotic area was evaluated quantitatively using an automated image analyzer, which determined the area occupied by Azan staining-positive interstitial tissue described previously [31,32]. Data were analyzed using Image-Pro plus software (Media Cybernetics Inc., Bethesda, MD, USA).

Glomerular podocyte injury was evaluated by immunohistochemical analysis of desmin and was performed as previously described [26,31].

### Dihydroethidium (DHE) immunofluorescence staining

 To investigate oxidative stress in the brain and kidney tissues, DHE immunofluorescence staining was performed as previously described [26]. Details are provided in the supplemental methods (Methods S1). All of the morphometric measurements were performed in a blinded manner to avoid any bias.

### NADPH oxidase activity

NADPH oxidase-derived superoxide anion () generation was measured using lucigenin-enhanced chemiluminescence, as described previously [33]. Details are provided in the supplemental methods (Methods S1).

### Real-time RT-PCR

 The mRNA expression in brain and renal cortical tissues were analyzed by RT-PCR using a LightCycler FastStart DNA Master SYBR Green I kit and an ABI Prism 7000 Sequence Detection System (Applied Biosystems, Foster City, USA) as previously described [26,31]. The oligonucleotide primer sequences for mice β-actin, claudin-5, occludin, zona occludin (ZO-1), p47phox, gp91phox, p22phox, alpha-smooth muscle actin (α-SMA) and type 1 collagen are listed in Table S1. All data are expressed as the relative difference in expression compared with C57BL6 controls after normalization for β-actin expression.

### Other analytical procedure

Plasma and urine 8-hydroxy-2’-deoxyguanosine (8-OHdG) (New 8-OHdG Check, JaICA, Shizuoka, Japan), plasma level of non-esterified fatty acid (NEFA), triglyceride (TG), total cholesterol (TCho) (all kits from Wako Co.Ltd., Osaka, Japan), and insulin (Rat Insulin ELISA kit; Shibayagi, Gunma, Japan) were measured using commercially available kits.

### Statistical analyses

 All values are presented as means ± S.E.M.. Statistical comparisons of differences among groups were performed using one-way repeated-measures analysis of variance (ANOVA), followed by the Newman-Keuls *post hoc* test. Values of *P* < 0.05 were considered statistically significant.

## Results

### SBP, body weight, PPBG and plasma lipid profiles

**Figure 1:**
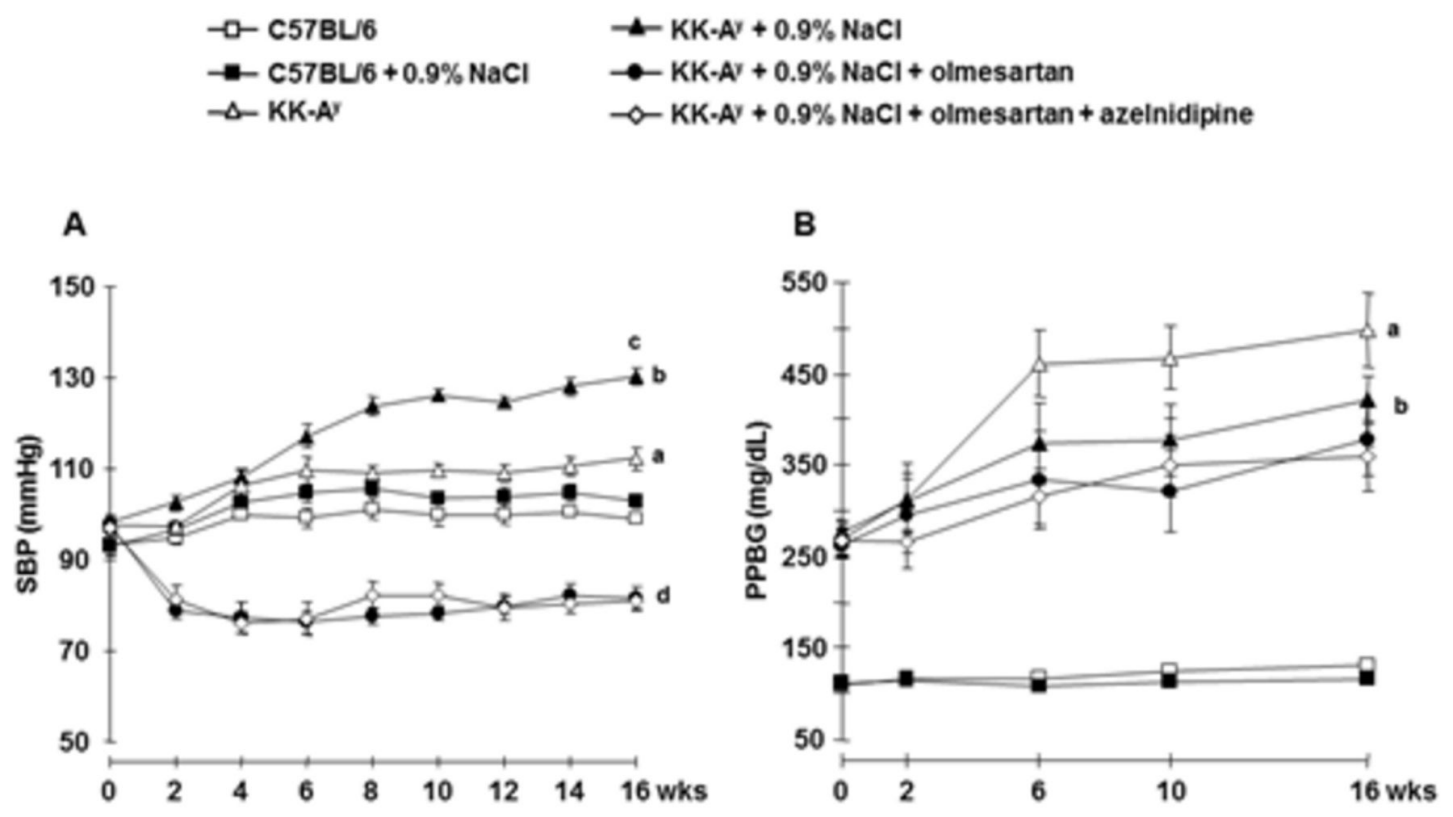
Systolic blood pressure (SBP) measured by tail-cuff plethysmography and postprandial blood glucose (PPBG) profiles. **A**, Saline-drinking KK-A^y^ mice showed hypertension, which was attenuated to similar levels by olmesartan, and the combination of olmesartan plus azelnidipine. **B**, None of the treatments significantly altered PPBG levels (n=11 in each group). ***^a^*** *P* < 0.05 vs. C57BL/6, ^b^ *P* < 0.05 vs. C57BL/6 + 0.9% NaCl, ^c^ *P* < 0.05 vs. KK-A^y^, ^d^ *P* < 0.05 vs. KK-A^y^ + 0.9% NaCl.

 During the 16 week treatment period, type 2 diabetic KK-A^y^ mice showed elevated SBP compared with that in age-matched C57BL6 or C57BL6 + 0.9% NaCl mice (Figure 1A). KK-A^y^ + 0.9% NaCl mice exhibited further SBP elevation compared with KK-A^y^ mice. Treatment with olmesartan, olmesartan + azelnidipine resulted in similarly reduced SBP in KK-A^y^ + 0.9% NaCl mice (Figure 1A).

At the end as well as during the experimental period, KK-A^y^ mice exhibited higher body weight (details are shown in Figure S1 and Table 1 respectively) and PPBG levels (details are shown in Figure 1B and Table 1 respectively) compared with C57BL6 mice. Compared with KK-A^y^ mice, KK-A^y^ + 0.9% NaCl mice demonstrated a tendency for lower PPBG levels, although this effect was not statistically significant. Furthermore, there were no significant differences in body weight or PPBG levels between KK-A^y^, KK-A^y^ + 0.9% NaCl, KK-A^y^ + 0.9% NaCl + olmesartan, and KK-A^y^ + 0.9% NaCl olmesartan + azelnidipine mice (Figure S1, and Figure 1B). At the end of experiment, KK-A^y^ mice exhibited elevated plasma insulin level compared with C57BL6 mice and was unaffected by saline drinking (Table 1). Treatment with olmesartan attenuated the increase in plasma insulin level in KK-A^y^ + 0.9%NaCl mice. Further reduction was observed in olmesartan + azelnidipine treated mice (Table 1).

**Table 1:** Parameters at the end of the experiment.

	Bwt (g)	PPBG (mg/dL)	Plasma Insulin (ng/mL)	TG (mg/dL)	TCho (mg/dL)	NEFA (mEq/L)
C57BL6	28.83±0.38	130.70±4.79	0.22±0.02	44.33±4.17	76.00±10.58	0.90±0.01
C57BL6+0.9% NaCl	27.80±0.49	116.10±3.68	0.23±0.02	39.20±7.93	87.00±5.11	0.88±0.02
KK-A**^*y*^**	45.58±1.14**^*a*^**	498.30±40.17 **^*a*^**	1.98±0.08 **^*a*^**	430.80±46.36 **^*a*^**	129.60±8.25 **^*a*^**	1.14±0.20
KK-A^y^+0.9% NaCl	44.63±1.16**^*b*^**	422.10±25.86 **^*b*^**	1.91±0.17 **^*b*^**	438.40±50.11 **^*b*^**	130.80±8.52 **^*b*^**	1.17±0.27
KK-A^y^+0.9% NaCl +olmesartan	46.87±0.97	378.50±40.12	1.30±0.14***^c^***,***^d^***	326.50±55.34 ^***c***,***d***^	95.50±7.41 ^***c***,***d***^	0.92±0.10
KK-A^y^+0.9% NaCl +olmesartan+azelnidipine	46.97±1.88	360.20±39.41	0.97±0.03**^*e*^**	205.00±35.04**^*e*^**	78.00±5.01**^*e*^**	0.96±0.03

Bwt; body weight, PPBG; post prandial blood glucose, TG; triglyceride, TCho; total cholesterol, NEFA; Non-esterified fatty acid. ***^a^*** *P* < 0.05 vs. C57BL/6, ***^b^*** *P* < 0.05 vs. C57BL/6 + 0.9% NaCl, ***^c^*** *P* < 0.05 vs. KK-A**^*y*^**, ***^d^*** *P* < 0.05 vs. KK-A^y^ + 0.9% NaCl, ***^e^*** *P* < 0.05 vs. KK-A^y^ + 0.9% NaCl + olmesartan.

KK-A^y^ mice exhibited elevated plasma TG, TCho levels compared with C57BL6 mice and was unaffected by saline drinking (Table 1). Treatment with olmesartan attenuated the increase in plasma TG and TCho levels in KK-A^y^ + 0.9%NaCl mice. Further reduction was observed in olmesartan + azelnidipine treated mice. However, plasma NEFA level was similar among the groups and none of the treatment significantly affects plasma NEFA level (Table 1).

### Cognitive function

**Figure 2:**
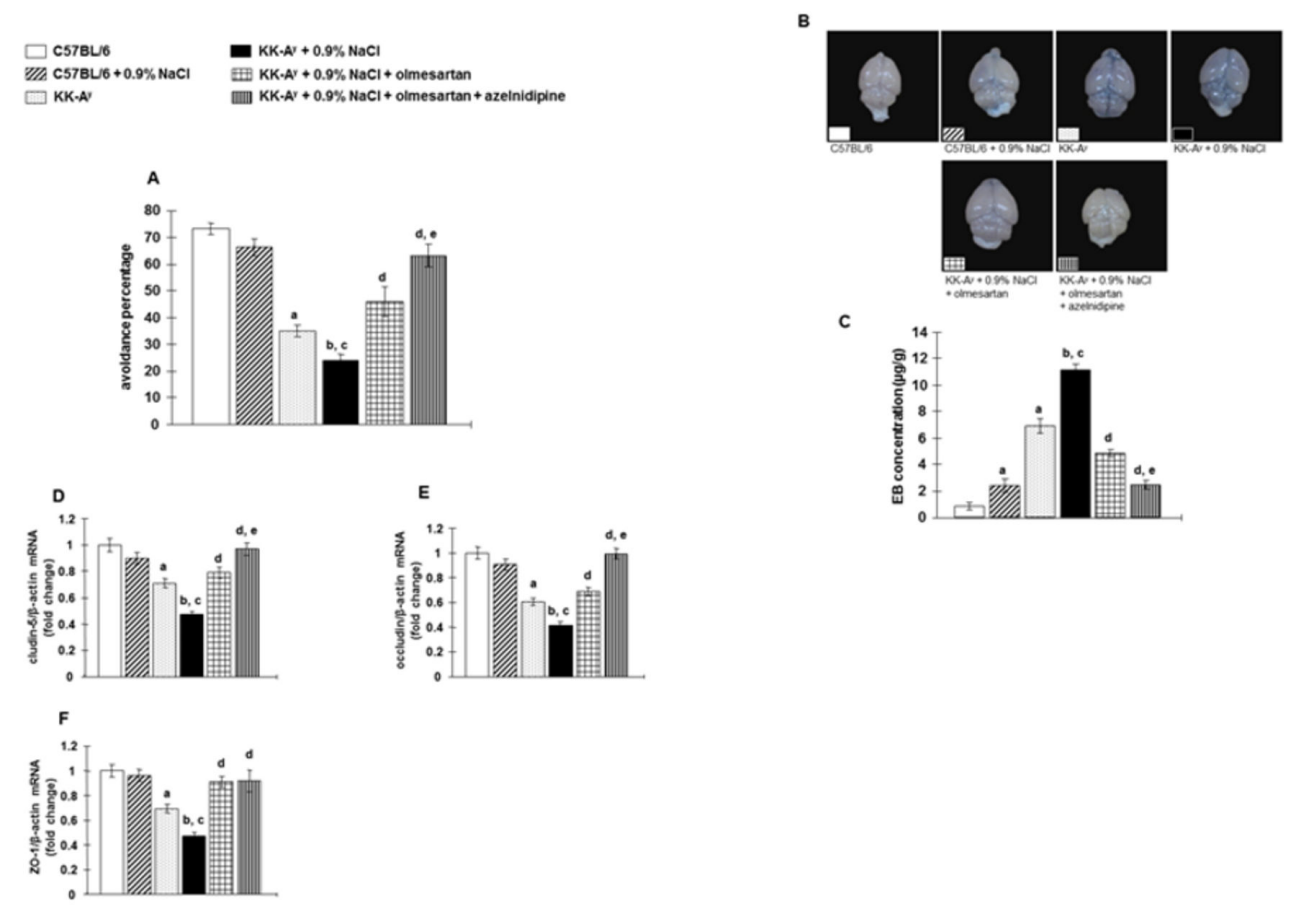
Evaluation of cognitive function, blood-brain barrier (BBB) leakage and mRNA expression of tight junction (TJ) associated proteins by passive avoidance test, Evans blue (EB) dye extravasation and by real-time RT-PCR (reverse transcription-polymerase chain reaction) analyses, respectively. **A**, Saline-drinking KK-A^y^ mice showed cognitive impairment, which was markedly improved by olmesartan. However, the combination of olmesartan plus azelnidipine prevented cognitive impairment (n=11 in each group). **B**, Representative macroscopic image of the pattern of EB dye extravasation (dark blue areas). The background was modified to black without any editing to clarify the picture. **C**, Quantification of EB dye concentration in whole brain tissue (n=6 in each group). Saline-drinking KK-A^y^ mice showed widespread EB dye extravasation in brain tissue. Olmesartan treatment showed a marked reduction in the EB dye concentration. However, the combination of olmesartan plus azelnidipine completely inhibited widespread EB dye extravasation in brain tissue. **D**–**F**, Saline-drinking KK-A^y^ mice showed downregulated mRNA expression of TJ associated proteins such as cludin-5, occludin, and zona occludin-1 (ZO-1) (n=8 in each group). Olmesartan markedly prevented the downregulated mRNA expression of TJ associated proteins. However, the combination of olmesartan plus azelnidipine completely prevented this downregulated mRNA expression of TJ associated proteins to levels similar to those in C57BL6 mice. ***^a^*** *P* < 0.05 vs. C57BL/6, ^b^ *P* < 0.05 vs. C57BL/6 + 0.9% NaCl, ^c^ *P* < 0.05 vs. KK-A^y^, ^d^ *P* < 0.05 vs. KK-A^y^ + 0.9% NaCl, ^e^ *P* < 0.05 vs. KK-A^y^ + 0.9% NaCl + olmesartan.

 Cognitive function was evaluated in passive avoidance tests [28]. Type 2 diabetic KK-A^y^ mice exhibited lower scores in passive avoidance tests compared with C57BL6 mice (Figure 2A). Furthermore, KK-A^y^ + 0.9% NaCl mice exhibited lower scores in passive avoidance tests compared with KK-A^y^ mice. Treatment of KK-A^y^ + 0.9% NaCl mice with olmesartan improved passive avoidance scores. In contrast, olmesartan + azelnidipine greatly improved cognitive function to a similar level as that observed in control C57BL6 mice (Figure 2A).

### BBB leakage and tight junction (TJ) associated proteins mRNA expression

 BBB leakages were examined by the EB dye extravasations technique [34]. Typical macroscopic observations for the EB dye extravasation are shown in Figure 2B. Saline-drinking resulted in increased EB dye content of brain tissues in both C57BL6 and KK-A^y^ mice (Figure 2C). Treatment of KK-A^y^ mice + 0.9% NaCl mice with olmesartan resulted in a significant reduction in the EB dye content of brain tissues. Furthermore, treatment with olmesartan + azelnidipine almost completely inhibited this increase in the EB dye content of brain tissues in KK-A^y^ + 0.9% NaCl mice.

Compared with control C57BL6 mice, type 2 diabetic KK-A^y^ mice showed reduced brain tissue gene expression of TJ associated proteins, such as cludin-5, occludin, and ZO-1 (Figure 2D, E and F). Reductions in these genes levels were further enhanced in KK-A^y^ + 0.9% NaCl mice. Treatment with olmesartan significantly attenuated the reductions in gene expression of TJ associated proteins in KK-A^y^ + 0.9% NaCl mice, while these levels were still significantly lower than those in control C57BL6 mice. In contrast, treatment with olmesartan + azelnidipine completely prevented the reduction in gene expression of TJ associated proteins (Figure 2D, E and F).

### Brain tissues oxidative stress, mRNA levels of NADPH oxidase subunits and NADPH oxidase activity

**Figure 3:**
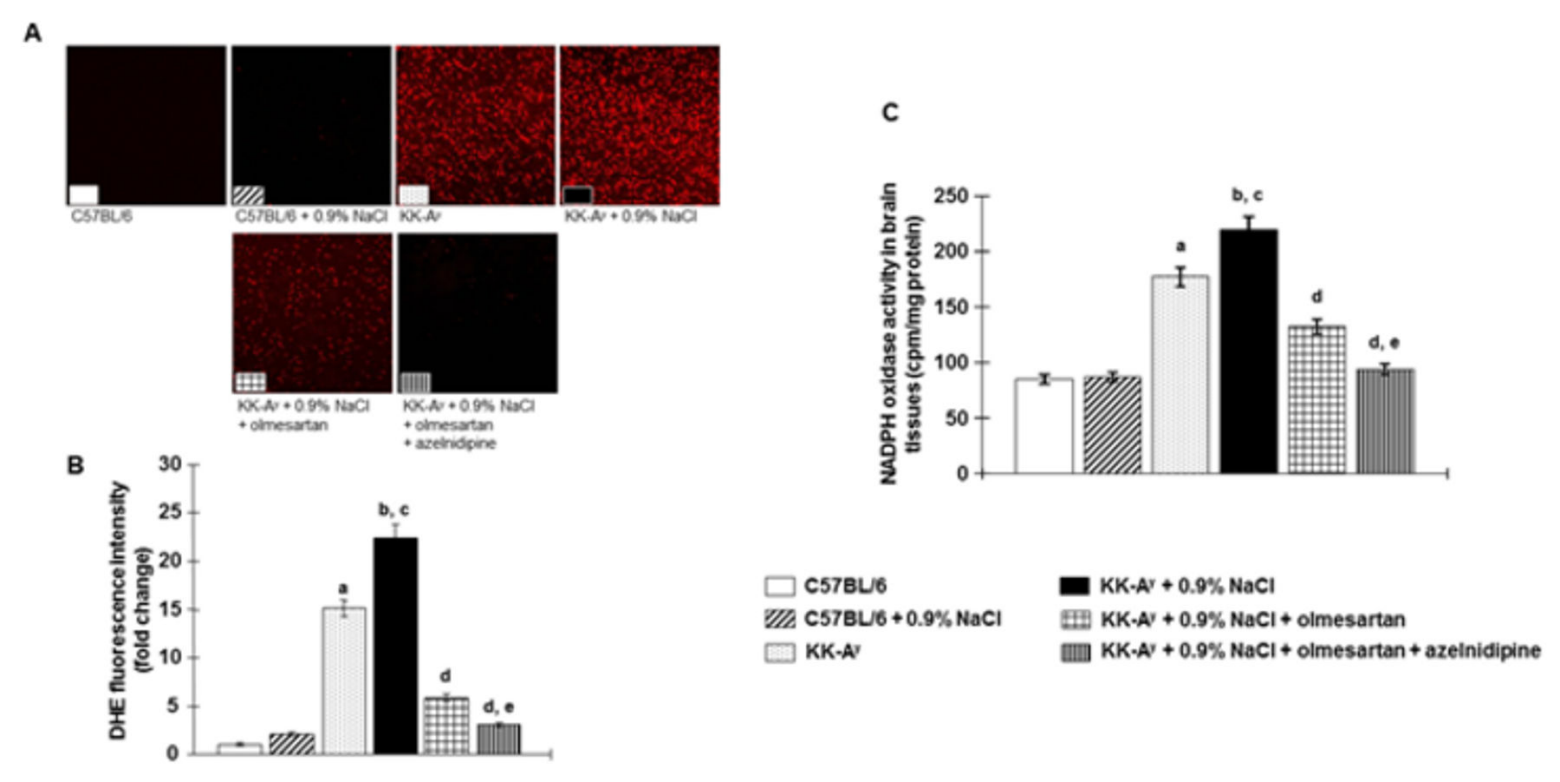
NADPH oxidase-dependent superoxide anion production in brain tissues were evaluated by dihydroethidium (DHE) immunofluorescence staining. **A**, Representative images of DHE immunofluorescence staining (original magnification, ×200). **B**, DHE fluorescence intensity (n=8 in each group). **C**, NADPH oxidase activity in homogenized brain tissues evaluated by lucigenin-enhanced chemiluminescence (n=6 in each group). Saline-dinking KK-A^y^ mice showed superoxide production in brain tissues and were associated with increased NADPH oxidase activity. Treatment with olmesartan markedly attenuated this superoxide production and suppresses NADPH oxidase activity. Furthermore, the combination of olmesartan plus azelnidipine completely ameliorated this NADPH oxidase-dependent superoxide production to a level similar to that in C57BL6 mice. ***^a^*** *P* < 0.05 vs. C57BL/6, ^b^ *P* < 0.05 vs. C57BL/6 + 0.9% NaCl, ^c^ *P* < 0.05 vs. KK-A^y^, ^d^ *P* < 0.05 vs. KK-A^y^ + 0.9% NaCl, ^e^ *P* < 0.05 vs. KK-A^y^ + 0.9% NaCl + olmesartan.

 We evaluated oxidative stress in the brain tissues using DHE immunofluorescence staining [26]. In KK-A^y^ mice, DHE staining was significantly increased in brain tissues compared with C57BL6 mice (Figure 3A and B). In KK-A^y^ mice, DHE staining was further increased by saline-drinking. Treatment with olmesartan attenuated the increase in DHE staining in KK-A^y^ + 0.9% NaCl mice. In these animals, the combination of olmesartan + azelnidipine normalized the DHE staining to a level similar to that of control C57BL6 mice.

Components of brain tissues such as NADPH oxidase, gp91phox and p47phox, were significantly upregulated in KK-A^y^ mice, and were further increased by saline-drinking (Figure S2A and B). Treatment with olmesartan significantly attenuated these increases in mRNA levels of gp91phox and p47phox in KK-A^y^ + 0.9% NaCl mice. In these animals, the combination of olmesartan + azelnidipine completely prevented this upregulation of mRNA expression to levels similar to those of control C57BL6 mice.

 In KK-A^y^ mice, NADPH oxidase activity was significantly increased in brain tissues compared with C57BL6 mice (Figure 3C) and was further increased by saline-drinking. Treatment with olmesartan suppresses the increase in NADPH oxidase activity in KK-A^y^ + 0.9% NaCl mice. In these animals, the combination of olmesartan + azelnidipine normalized the NADPH oxidase activity to a level similar to that of control C57BL6 mice.

### Albuminuria and glomerular podocyte injury

**Figure 4:**
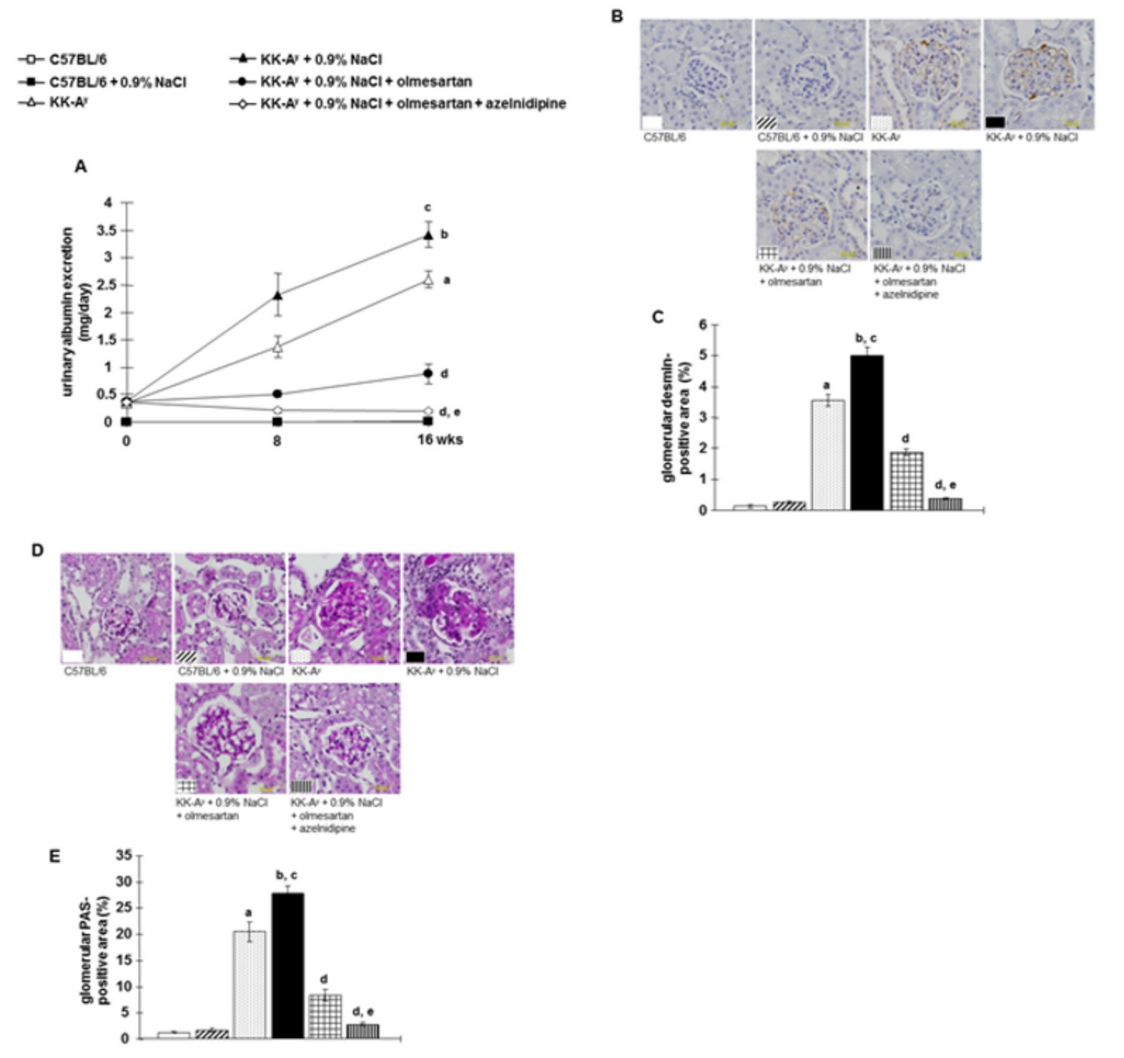
Albuminuria and glomerular podocyte injury. **A**, KK-A^y^ mice showed albuminuria, which was exacerbated by further increased saline intake (n=11 in each group). **B**, Glomerular podocyte injury was detected by desmin immunostaining. Representative desmin-stained images (scale bar shows the values), and **C**, the desmin-positive area as a percentage of the total glomerular area. KK-A^y^ mice showed larger desmin-positive areas (brown staining) in the glomeruli, which were further increased by saline intake. Olmesartan markedly prevented these changes. However, the combination of olmesartan plus azelnidipine almost completely abrogated albuminuria and completely prevented glomerular podocyte injury. **D**, Glomerular sclerosis was evaluated by examining periodic acid-Schiff (PAS) staining. Representative micrographs of PAS-stained renal sections (scale bar shows the values), and **E**, the PAS-positive area within the total glomerular area. KK-Ay mice exhibit severe glomerular sclerosis, which was further exacerbated by saline intake. Olmesartan markedly prevented glomerular sclerosis. However, the combination of olmesartan plus azelnidipine exhibited greater protective efficacy against glomerular sclerosis (n=7 in each group). ***^a^*** *P* < 0.05 vs. C57BL/6, ^b^ *P* < 0.05 vs. C57BL/6 + 0.9% NaCl, ^c^ *P* < 0.05 vs. KK-A^y^, ^d^ *P* < 0.05 vs. KK-A^y^ + 0.9% NaCl, ^e^ *P* < 0.05 vs. KK-A^y^ + 0.9% NaCl + olmesartan.

 KK-A^y^ mice showed overt albuminuria, which was further augmented by saline-drinking (Figure 4A). Glomerular podocyte injury determined by desmin immunostaining [35] was markedly increased (brown staining) in KK-A^y^ mice compared with control C57BL6 mice (Figures 4B and C). KK-A^y^ + 0.9% NaCl mice showed further increased desmin staining in glomeruli. Treatment with olmesartan attenuated the development of albuminuria and podocyte injury in KK-A^y^ + 0.9% NaCl mice. In contrast, the combination of olmesartan + azelnidipine almost completely abrogated albuminuria and completely prevented glomerular podocyte injury in KK-A^y^ + 0.9% NaCl mice.

### Glomerular sclerosis and renal interstitial fibrosis

 Significant glomerular sclerosis was observed in KK-A^y^ mice, which was further aggravated by saline-drinking (Figures 4D and E). Treatment with olmesartan attenuated glomerular sclerosis in KK-A^y^ + 0.9% NaCl mice. In these animals, greater protective efficacy was elicited by combination therapy with olmesartan + azelnidipine.

**Figure 5:**
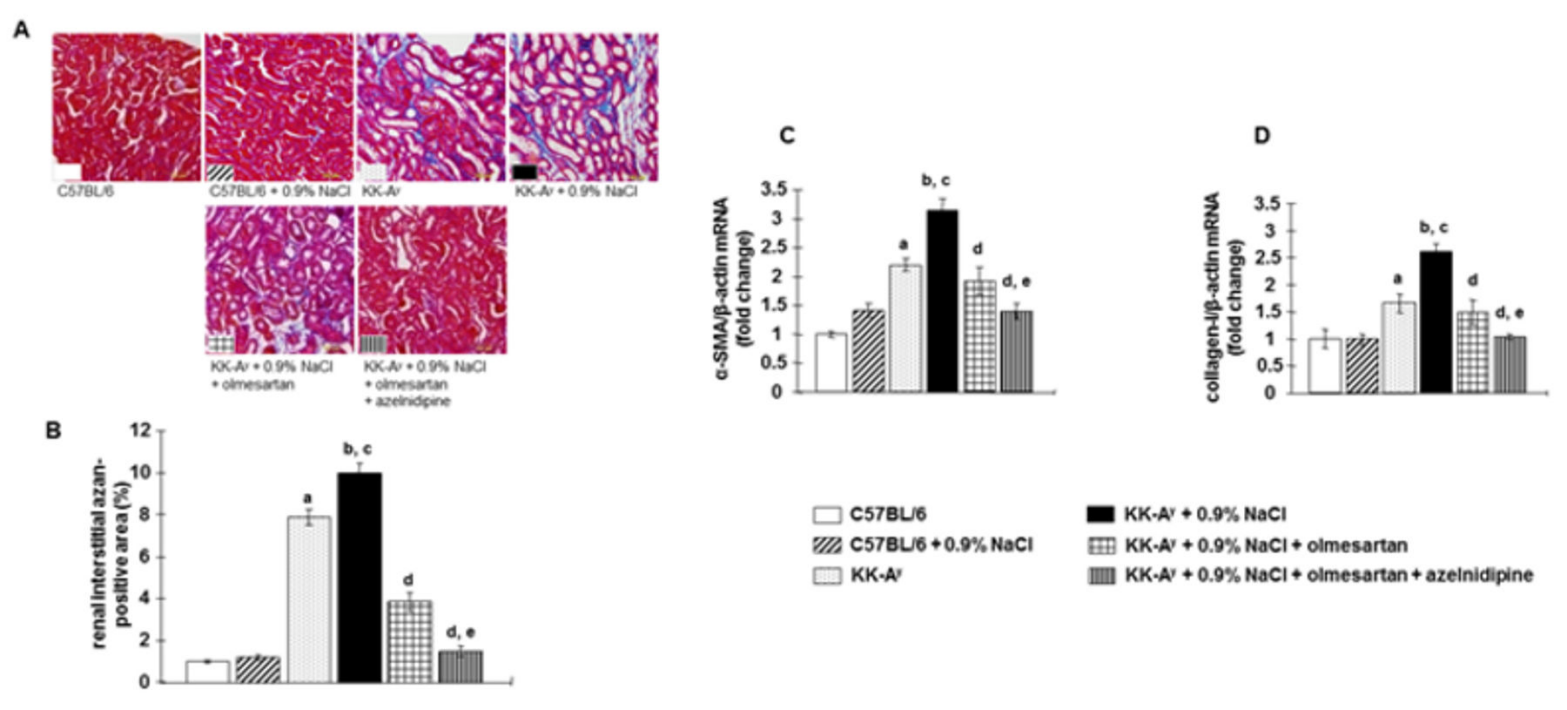
Renal tubulointerstitial fibrosis was detected by Azan staining. **A**, Representative micrographs of Azan-stained renal sections (scale bar shows the values), and **B**, the Azan-positive area. **C**, Gene expression of α-smooth muscle actin (α-SMA), and **D**, type 1 collagen. KK-A^y^ mice exhibited increased Azan-positive area (blue staining) in the tubulointerstitium and profibrotic gene expression in renal cortical tissues, which were further exacerbated by saline intake. Olmesartan markedly prevented these changes. However, the combination of olmesartan plus azelnidipine exhibited greater protective efficacy (n=7 in each group). ***^a^*** *P* < 0.05 vs. C57BL/6, ^b^ *P* < 0.05 vs. C57BL/6 + 0.9% NaCl, ^c^ *P* < 0.05 vs. KK-A^y^, ^d^ *P* < 0.05 vs. KK-A^y^ + 0.9% NaCl, ^e^ *P* < 0.05 vs. KK-A^y^ + 0.9% NaCl + olmesartan.

Renal tubulointerstitial fibrosis was markedly increased in KK-A^y^ mice (blue staining) (Figures 5A and B). In addition, KK-A^y^ mice showed upregulated expression of profibrotic genes such as α-SMA and collagen-1 in renal cortical tissues (Figures 5C and D). These detrimental changes were more marked following saline-drinking in KK-A^y^ mice. Treatment with olmesartan markedly attenuated tubulointerstitial fibrosis and upregulation of profibrotic gene expression in KK-A^y^ + 0.9% NaCl mice. Interestingly, greater protective efficacy was elicited by combination therapy with olmesartan + azelnidipine.

### Renal tissues oxidative stress, mRNA levels of NADPH oxidase subunits and NADPH oxidase activity

**Figure 6:**
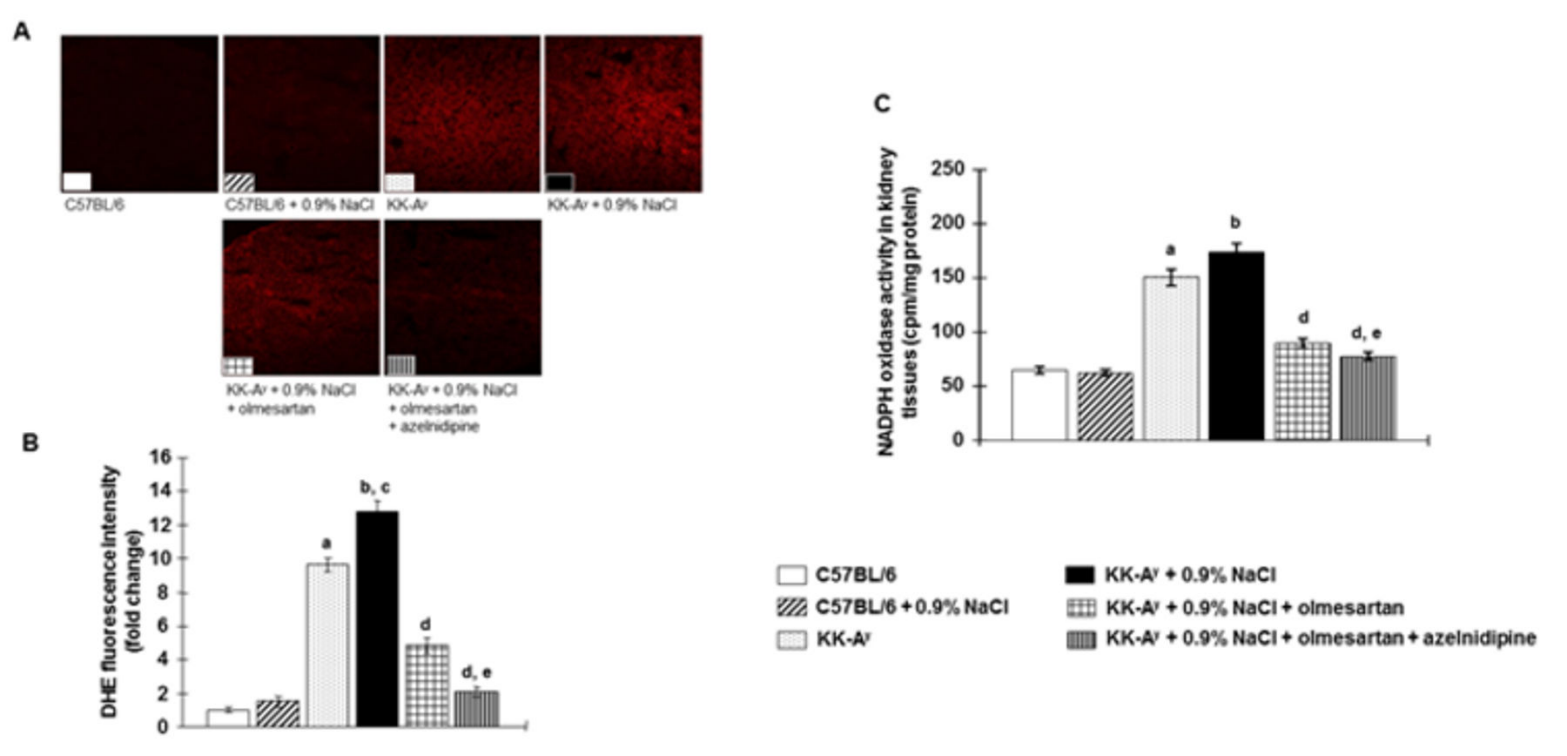
NADPH oxidase-dependent superoxide anion production in kidney tissues. **A**, Representative images of dihydroethidium (DHE) staining (original magnification, ×100). **B**, DHE fluorescence intensity (n=8 in each group). **C**, NADPH oxidase activity in homogenized renal cortical tissues (n=6 in each group). KK-A^y^ mice showed increased superoxide production in kidney tissues and were associated with increased NADPH oxidase activity, which were further increased by saline intake. Olmesartan markedly prevented superoxide production and NADPH oxidase activity in kidney tissues. Interestingly, the combination of olmesartan plus azelnidipine completely prevented NADPH oxidase-dependent superoxide production in kidney tissues. ***^a^*** *P* < 0.05 vs. C57BL/6, ^b^ *P* < 0.05 vs. C57BL/6 + 0.9% NaCl, ^c^ *P* < 0.05 vs. KK-A^y^, ^d^ *P* < 0.05 vs. KK-A^y^ + 0.9% NaCl, ^e^ *P* < 0.05 vs. KK-A^y^ + 0.9% NaCl + olmesartan.

 In KK-A^y^ mice, DHE staining was significantly increased in kidney tissues compared with that in C57BL6 mice (Figures 6A and B). DHE staining was further increased by saline-drinking in KK-A^y^ mice. Treatment with olmesartan markedly attenuated the increase in DHE staining in KK-A^y^ + 0.9% NaCl mice. In these animals, the combination of olmesartan + azelnidipine almost completely abolished DHE staining in the kidney.

Increased DHE staining in renal tissues was associated with upregulation of NADPH oxidase components, gp91phox and p22phox, in KK-A^y^ mice, which were further upregulated by saline-drinking (Figure S3A and B). Olmesartan, but not hydralazine, attenuated the increases in expression of these genes in KK-A^y^ + 0.9% NaCl mice. In these animals, increased expression of these genes was completely prevented by combination therapy with olmesartan + azelnidipine.

In KK-A^y^ mice, NADPH oxidase activity was significantly increased in kidney tissues compared with C57BL6 mice and was further non-significantly increased by saline-drinking (Figure 6C). Treatment with olmesartan suppresses the increase in NADPH oxidase activity in KK-A^y^ + 0.9% NaCl mice. Interestingly, greater NADPH oxidase activity suppressive efficacy was elicited by combination therapy with olmesartan + azelnidipine compared to olmesartan alone.

### Plasma and urine 8-OHdG

**Figure 7:**
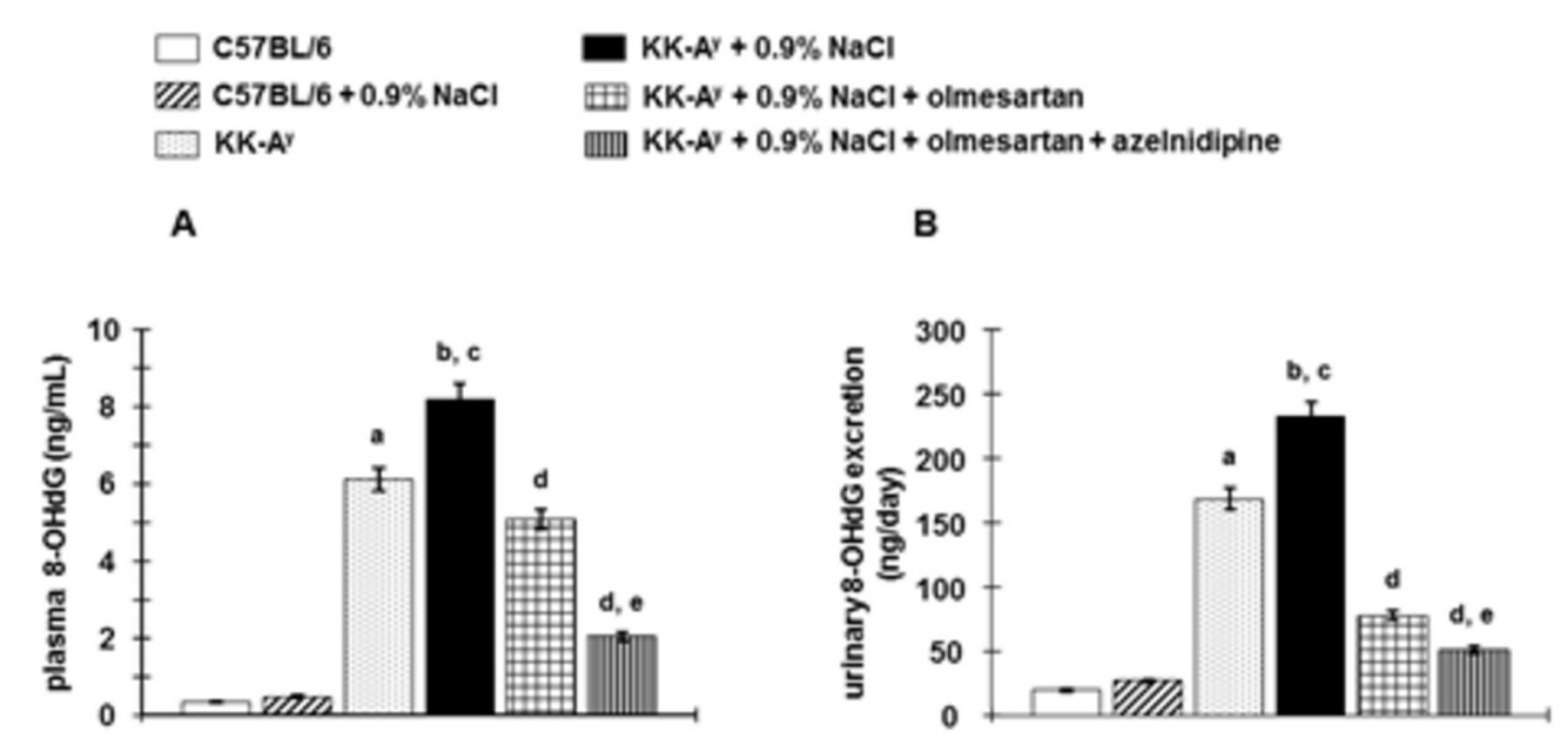
Systemic oxidative stress was evaluated by measuring plasma and urine 8-hydroxy-2’-deoxyguanosine (8-OHdG). **A**, KK-A^y^ mice showed elevated plasma 8-OHdG, and **B**, increased urinary excretion of 8-OHdG, which were exacerbated by further increased saline intake (n=6 in each group). Treatment with olmesartan suppresses the elevated plasma and urinary excretion of 8-OHdG. Furthermore, the combination of olmesartan plus azelnidipine exhibited greater suppressive efficacy (n=8 in each group). ***^a^*** *P* < 0.05 vs. C57BL/6, ^b^ *P* < 0.05 vs. C57BL/6 + 0.9% NaCl, ^c^ *P* < 0.05 vs. KK-A^y^, ^d^ *P* < 0.05 vs. KK-A^y^ + 0.9% NaCl, ^e^ *P* < 0.05 vs. KK-A^y^ + 0.9% NaCl + olmesartan.

Systemic oxidative stress was evaluated by measuring plasma 8-OHdG and urinary excretion of 8-OHdG. KK-A^y^ mice exhibited elevated plasma 8-OHdG and increased urinary excretion of 8-OHdG compared with C57BL6 mice and were further increased by saline drinking (Figure 7A and B). Treatment with olmesartan suppresses the increase in plasma 8-OHdG and reduced urinary excretion of 8-OHdG in KK-A^y^ + 0.9%NaCl mice. Interestingly, further reduction was observed in olmesartan + azelnidipine treated mice compared to olmesartan alone. 

## Discussion

 Both clinical and basic evidence indicates that the combination of an ARB and a CCB is protective against cerebrovascular and renal injury. In the present study, we showed that treatment with the ARB, olmesartan, effectively attenuated cognitive impairment and BBB disruption as well as renal injury in salt-treated type 2 diabetic mice. Interestingly, co-administration of suppressive dose of the CCB, azelnidipine, significantly exaggerated the protective effects of olmesartan. These results are in accordance with previous studies by Horiuchi and colleagues who showed that a suppressive dose of azelnidipine enhanced the inhibitory action of olmesartan on ischemic brain damage [36] and mechanical vascular injury in mice [37]. Interestingly, they also reported that the synergistic actions of olmesartan and azelnidipine were observed in the inhibition of Ang II-induced cell proliferation in cultured vascular smooth muscle cells [37,38].

Impaired BBB function has been shown to contribute to the pathophysiology of several brain diseases, including cognitive disorders [39]. We and others have previously reported that high salt exacerbates cognitive impairment, and BBB leakage, with decreased gene expression of TJ associated proteins such as occludin, claudin-5 and zona occluding [28,40]. The present study showed that these effects were also observed in KK-A^y^ mice and were further exaggerated by high salt intake in type 2 diabetic KK-A^y^ mice. In agreement with our previous studies [41,42], type 2 diabetic KK-A^y^ mice showed renal injury characterized by glomerular podocyte injury leading to albuminuria, glomerular sclerosis and tubulointerstitial fibrosis. Similar to the effects observed on cognitive impairment and BBB disruption, these renal injuries were further aggravated by high salt intake in KK-A^y^ mice. Collectively, these data suggest that high salt intake aggravates cerebrovascular-renal injuries in type 2 diabetes.

Previous studies have shown that a loss of memory function is associated with increased oxidative stress in the brain and that antioxidative treatment reversed the behavioral changes [43]. There is also clinical evidence of increased oxidative damage in subjects with mild cognitive impairment [44]. Dobrian et al. [45] reported that high salt intake induces increased vascular oxidative stress in rats, suggesting the role of oxidative stress in vascular injury. Other clinical studies have also highlighted that increased oxidative stress may contribute to the pathogenesis of diabetic complications including nephropathy [46-48]. The present study showed that augmentation of superoxide anion production in brain and kidney tissues was associated with upregulated expression of both membrane and cytosolic components of NADPH oxidase as well as NADPH oxidase activity in type 2 diabetic KK-A^y^ mice. Moreover, high salt intake in KK-A^y^ mice further increased superoxide anion production, NADPH oxidase subunit expression and NADPH oxidase activity in brain and kidney tissues. Furthermore, these changes were associated with elevated systemic oxidative stress. These results suggest that augmentation of NADPH oxidase-dependent local and systemic oxidative stress plays an important role in the pathogenesis of cerebrovascular-renal injuries in type 2 diabetic subjects with high salt intake.

The mechanism of the synergistic or beneficial effects of the combined use of dihydropyridine, CCB and ARB is not yet clear; however, both clinical and basic studies have highlighted the potential roles of their antioxidative properties [36,37,46,49]. In the present study, we found that co-administration of suppressive doses of azelnidipine with olmesartan further reduced NADPH oxidase-dependent oxidative stress compared with those mediated by olmesartan alone. These data are consistent with previous studies demonstrating that dihydropyridine CCBs elicit antioxidative activity not only by blocking the AT_1_ receptor-mediated signaling pathway, but also via other mechanisms [25,36,50]. However, the precise molecular mechanism by which CCB enhances the inhibitory effects of an ARB on NADPH oxidase-dependent oxidative stress is not yet clear. 

A possible role of ROS in the regulation of TJ-associated protein has been reported; however, the precise mechanisms are unclear. Several studies have shown that ROS alters blood-brain barrier integrity, which is associated with disappearance in gene expressions of TJ-associated protein [51,52] as observed in the present study. In the present study, brain tissue mRNA levels of TJ-associated proteins in ARB- and ARB+CCB-treated mice were significantly increased compared to untreated animals. Furthermore, these effects of ARB and ARB+CCB were associated with a reduction in ROS levels in brain tissue. We speculate that antioxidative effects of ARB and ARB+CCB may contribute, at least in part, to changes in mRNA levels of TJ-associated proteins. Further studies are required to determine the precise mechanisms by which ROS suppress TJ-associated protein. The present study showed that a combination of suppressive doses of azelnidipine with olmesartan reduced blood pressure to levels similar to those observed with olmesartan alone. However, a combination of suppressive doses of azelnidipine with olmesartan showed greater cerebrovascular-renal protective efficacy compared to olmesartan alone. This result further suggests that the beneficial effects of co-administration of suppressive doses of azelnidipine and olmesartan are independent of their antihypertensive activity in this pathophysiological condition.

In conclusion, the present data support our hypothesis postulated on the basis of the results of clinical studies that show that a CCB enhances the cerebrovascular-renal protective effects of an ARB, independent of blood pressure reduction in type 2 diabetes. Co-administration of a CCB and an ARB may be an effective therapeutic strategy for cerebrovascular-renal injury in type 2 diabetes.

## Supporting Information

methods S1 (DOCX)

Table S1 (DOCX)

Figure S1 **Body weight changes during the experimental period.** KK-A^y^ mice showed higher body weight compared to C57BL mice. However, none of the treatments affected body weight gains in KK-Ay + 0.9% NaCl mice (n=11). ***^a^*** *P* < 0.05 vs. C57BL/6, ^b^ *P* < 0.05 vs. C57BL/6 + 0.9% NaCl.
(TIF)

Figure S2 **NADPH oxidase subunits gene expression in brain tissues analyzed by RT-PCR.** NADPH oxidase subunits gp91phox (**A**) and p47phox (**B**) mRNA levels in whole brain tissues. Saline-dinking KK-A^y^ mice showed upregulation of NADPH oxidase subunit mRNA levels in brain tissues, which were attenuated by treatment with olmesartan. Furthermore, the combination of olmesartan plus azelnidipine completely prevented these changes resulting in levels similar to that in C57BL6 mice (n=8). ***^a^*** *P* < 0.05 vs. C57BL/6, ^b^ *P* < 0.05 vs. C57BL/6 + 0.9% NaCl, ^c^ *P* < 0.05 vs. KK-A^y^, ^d^ *P* < 0.05 vs. KK-A^y^ + 0.9% NaCl, ^e^ *P* < 0.05 vs. KK-A^y^ + 0.9% NaCl + olmesartan.
(TIF)

Figure S3 **NADPH oxidase subunits gene expression in kidney tissues analyzed by RT-PCR.** NADPH oxidase subunits gp91phox (**A**) and p22phox (**B**) mRNA levels in renal cortical tissues. In saline-dinking KK-A^y^ mice, superoxide production in renal tissues was associated with upregulation of NADPH oxidsase subunit genes expression. Treatment with olmesartan markedly attenuated these changes. Furthermore, the combination of olmesartan plus azelnidipine completely prevented these changes resulting in levels similar to that in C57BL6 mice (n=8). ***^a^*** *P*<0.05 vs. C57BL/6, ^b^ *P*<0.05 vs. C57BL/6 + 0.9% NaCl, ^c^ *P*<0.05 vs. KK-A^y^, ^d^ *P*<0.05 vs. KK-A^y^ + 0.9% NaCl, ^e^ *P*<0.05 vs. KK-A^y^ + 0.9% NaCl + olmesartan.
(TIF)
