# Supplementary material for: Calcium Channel Blocker Enhances Beneficial Effects of an Angiotensin II AT1 Receptor Blocker against Cerebrovascular-Renal Injury in type 2 Diabetic Mice
Source: PLoS One. 2013 Dec 10;8(12):e82082. doi: 10.1371/journal.pone.0082082 (PMC3858271; doi:10.1371/journal.pone.0082082)
Supplement: methods S1 — (DOCX) [file pone.0082082.s001.docx]

**SUPPLEMENTAL MATERIALS**

**Calcium channel blocker enhances beneficial effects of an angiotensin II AT_1_ receptor blocker against cerebrovascular-renal injury in type 2 diabetic mice**

Kazi Rafiq^1^, Shamshad J. Sherajee^1^, Hirofumi Hitomi^1^, Daisuke Nakano^1^, Hiroyuki Kobori^1^, Koji Ohmori^2^, Hirohito Mori^3^, Hideki Kobara^3^, Tsutomu Masaki^3^, Masakazu Kohno^2^, Akira Nishiyama^1^

^1^Department of Pharmacology, ^2^Cardiorenal and Cerebrovascular Medicine, and ^3^Gastroenterology, Faculty of Medicine, Kagawa University, Kagawa, Japan

**Running title:** CCB and ARB in cerebrovascular-renal injury

**All correspondence to:**

Kazi Rafiq, PhD

Department of Pharmacology, Faculty of Medicine, Kagawa University,

1750-1 Ikenobe, Miki-cho, Kita-gun, Kagawa 761-0793, Japan

Phone: +81 87 891 2125, Fax: +81 87 891 2126

E-mail: [krafiq73@yahoo.com](mailto:krafiq73@yahoo.com); [kazir73@med.kagawa-u.ac.jp](mailto:kazir73@med.kagawa-u.ac.jp)

**Materials and methods**

**Passive avoidance test**

A shuttle avoidance cage (32 × 12 × 15 cm; Melquest, Toyama, Japan) and an isolation cabinet (48 × 42 × 37 cm; Melquest, Toyama, Japan) [[1](#_ENREF_1)] were used. The shuttle avoidance cage was divided into equal-sized chambers by a divider with a door of sufficient size to allow movement between chambers. The floor of the shuttle box consisted of stainless steel rods. Scrambled shocks were delivered by a shock generator (SG-200, Melquest, Toyama, Japan) [[1](#_ENREF_1)]. Mice were placed individually into a chamber and given 20 inescapable electric shocks (0.3 mA) of 3 sec duration at intervals of 2 sec. A buzzing tone followed by a light signal were presented during the first 10 sec of each trial. If there was no avoidance response within this period, the tone remained on and a 0.3 mA shock was delivered for a duration of 3 sec through the grid floor. In the case of no escape response within this period, both the tone and shock were automatically terminated. The inter-trial interval was 10 sec. The number of escape failures was recorded. Escape failure was defined as a non-crossing response during shock delivery. Finally, cognitive function was determined as the percentage avoidance rate. In this study, passive avoidance tests were performed in the morning once a week for the first 14 weeks and on alternate days for the last 2 weeks of the study.

**Evans Blue (EB) assay**

EB dye (2%; 4 ml/kg body weight) was injected intravenously through tail vein in conscious mice (*n* = 5 per group) and allowed to circulate for 30 min [[2](#_ENREF_2),[3](#_ENREF_3)]. Mice were then transcardially perfused with 40 ml of chilled saline for 10 min to remove intravascular EB dye. After decapitation, the brain was removed frozen in liquid nitrogen and stored at -80°C. Each brain was weighed, homogenized in 1.25 ml phosphate buffered saline (PBS) and centrifuged (10 min, 14,000 ×*g*, 4°C). The supernatant was collected and equal amounts of 50% trichloroacetic acid was added to precipitate protein. Samples were later cooled for 30 min followed by centrifugation (10 min, 14,000×*g*, 4°C). The concentration of EB dye in the supernatant was measured at 620 nm using a spectrophotometer (Microplate Reader; SH-9000Lab, CORONA Electric Co., Ltd., Ibaragi, Japan) [[4](#_ENREF_4)]. EB dye concentration was expressed as µg/mg of brain tissue against a standard curve.

**Immunohistochemical detection of desmin**

Immunohistochemical detection of desmin was performed using the Histofine Simple Stain MAX-PO MULTI (Nichirei Biosciences, Tokyo, Japan) [[5](#_ENREF_5),[6](#_ENREF_6)]. Deparaffinized sections were incubated with 0.1% hydrogen peroxide for 10 min to block endogenous enzymes. After blocking, sections were incubated with primary antibodies (mouse anti-human desmin monoclonal antibody, D33, 1:500, DAKOCytomation, Glostrup, Denmark) for 10 min at room temperature. Immunoreactivity was visualized by DAB (3, 3-diaminobenzidine tetrahydrochloride) substrate (DAKOCytomation); counterstaining was performed with hematoxylin (DAKOCytomation). Sections incubated without primary antibodies were used as controls. The histologic analysis was performed using Image-Pro plus software (Media Cybernetics, Bethesda, MD, USA).

**Dihydroethidium (DHE) staining**

Fresh brain and kidney tissues were embedded in OCT immediately after sacrifice and were subsequently cryosectioned (8-µm sections). The sections were incubated with 10 μmol/L DHE (Invitrogen, Carlsbad, CA, USA) in PBS at 37°C for 30 min in a light-protected humidified chamber to determine superoxide anion production in the tissues [[4](#_ENREF_4),[6](#_ENREF_6),[7](#_ENREF_7)]. Once DHE is oxidized to ethidium, it intercalates within DNA, staining the nucleus a fluorescent red. Images were obtained using a confocal laser-scanning fluorescence microscopy system (Radiance2100; Bio-Rad Laboratories, Hercules, CA, USA). All of the morphometric measurements were performed in a blinded manner to avoid any bias.

**NADPH Oxidase Activity**

Brain and kidney tissues were placed in chilled phosphate-buffered saline containing protease inhibitor and homogenized to prepare 10% tissue homogenate using micro homogenizing system (Micro Smash^TM^, TOMY MEDICO., LTD, Tokyo, Japan) at 4℃. Protein concentration of homogenates was measured using the Bradford protein assay kit (Bio-Rad Laboratories, Hercules, CA). After centrifuging, the supernatant was transferred into a glass test tube containing lucigenin (final concentration 5 μM in Krebs-HEPES buffer). Chemiluminescence was then recorded every 30 sec for 10 min with a luminescence reader (BLR-301, Aloka, Tokyo, Japan), and the readings in the last 5 min were averaged. After measurement of background lucigenin chemiluminescence, NADPH was added to a final concentration of 100 μM. Thereafter, chemiluminescence was recorded another 10 min, and the readings in the last 5 min were averaged. To verify if the lucigenin signal reflects
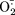
 generation, an NADPH oxidase inhibitor, diphenylene iodonium, was added at the end of measurements (final concentration, 10 μM). In all samples, diphenylene iodonium reduced NADPH-induced increases in chemiluminescence to background levels (data not shown). The differences between the values obtained before and after adding the NADPH were calculated, and the activity of NADPH oxidase was expressed as counts per minute per milligram of protein [[8](#_ENREF_8),[9](#_ENREF_9)].

**References**

1. Mogi M, Tsukuda K, Li JM, Iwanami J, Min LJ, et al. (2007) Inhibition of cognitive decline in mice fed a high-salt and cholesterol diet by the angiotensin receptor blocker, olmesartan. Neuropharmacology 53: 899-905.

2. Avtan SM, Kaya M, Orhan N, Arslan A, Arican N, et al. (2011) The effects of hyperbaric oxygen therapy on blood-brain barrier permeability in septic rats. Brain Res 1412: 63-72.

3. Manaenko A, Fathali N, Khatibi NH, Lekic T, Hasegawa Y, et al. (2011) Arginine-vasopressin V1a receptor inhibition improves neurologic outcomes following an intracerebral hemorrhagic brain injury. Neurochem Int 58: 542-548.

4. Rafiq K, Noma T, Fujisawa Y, Ishihara Y, Arai Y, et al. (2012) Renal sympathetic denervation suppresses de novo podocyte injury and albuminuria in rats with aortic regurgitation. Circulation 125: 1402-1413.

5. Miyata K, Ohashi N, Suzaki Y, Katsurada A, Kobori H (2008) Sequential activation of the reactive oxygen species/angiotensinogen/renin-angiotensin system axis in renal injury of type 2 diabetic rats. Clin Exp Pharmacol Physiol 35: 922-927.

6. Rafiq K, Nakano D, Ihara G, Hitomi H, Fujisawa Y, et al. (2011) Effects of mineralocorticoid receptor blockade on glucocorticoid-induced renal injury in adrenalectomized rats. J Hypertens 29: 290-298.

7. Fan YY, Kohno M, Nakano D, Hitomi H, Nagai Y, et al. (2009) Inhibitory effects of a dihydropyridine calcium channel blocker on renal injury in aldosterone-infused rats. J Hypertens 27: 1855-1862.

8. Nakano D, Hayashi T, Tazawa N, Yamashita C, Inamoto S, et al. (2005) Chronic hypoxia accelerates the progression of atherosclerosis in apolipoprotein E-knockout mice. Hypertens Res 28: 837-845.

9. Rahman M, Nishiyama A, Guo P, Nagai Y, Zhang GX, et al. (2006) Effects of adrenomedullin on cardiac oxidative stress and collagen accumulation in aldosterone-dependent malignant hypertensive rats. J Pharmacol Exp Ther 318: 1323-1329.
