# Supplementary material for: Calcium Channel Blocker Enhances Beneficial Effects of an Angiotensin II AT1 Receptor Blocker against Cerebrovascular-Renal Injury in type 2 Diabetic Mice
Source: PLoS One. 2013 Dec 10;8(12):e82082. doi: 10.1371/journal.pone.0082082 (PMC3858271; doi:10.1371/journal.pone.0082082)
Supplement: Table S1 — (DOCX) [file pone.0082082.s002.docx]

**Table S1.** The oligonucleotide primer sequences for real-time RT-PCR.

| Name |  | Sequences |
| --- | --- | --- |
| β-actin | sense | 5′-CCCGCGAGCACAGCTTCTTTG-3′ |
|  | antisense | 5′-ACATGCCGGAGCCGTTGTCGAC-3′ |
| Claudin-5 | sense | 5′-CTTTACGCGGTGTGCGGGCT-3′ |
|  | antisense | 5′-CACCGGCACCGTCGGATCAT-3′ |
| Occludin | sense | 5′-TGTCCGGCCGATGCTCTCTCA-3′ |
|  | antisense | 5′-CTGGGGGCGACGTCCATTTGT-3′ |
| ZO-1 | sense | 5′-ACTCACCACAAGCGCAGCCAC-3′ |
|  | antisense | 5′-TGCTGGGGTTGTTTCAGGCGAA-3′ |
| p47phox | sense | 5′-TCGACCATCCGCAACGCACA-3′ |
|  | antisense | 5′-CGGACGCTGTTGCGGCGATA-3′ |
| gp91phox | sense | 5′-AGGGGTTCCAGTGCGTGTTGC-3′ |
|  | antisense | 5′-TGTGGATGGCGGTGTGCAGTG-3′ |
| p22phox | sense | 5′-TCACTGGGGGCATCGTGGCT-3′ |
|  | antisense | 5′-TCGCTCCATGGTGGACCCCTT-3′ |
| α-SMA | sense | 5′-AAGCCCAGCCAGTCGCTGTCA-3′ |
|  | antisense | 5′-AGCCCAGAGCCATTGTCGCA-3′ |
| Type 1 collagen | sense | 5′-AATGGCACGGCTGTGTGCGA-3′ |
|  | antisense | 5′-AGCACTCGCCCTCCCGTCTT-3′ |

ZO-1; zona occludin, α-SMA; alpha-smooth muscle actin.
