# Supplementary figures and images for: Calcium Channel Blocker Enhances Beneficial Effects of an Angiotensin II AT1 Receptor Blocker against Cerebrovascular-Renal Injury in type 2 Diabetic Mice
Source: PLoS One. 2013 Dec 10;8(12):e82082. doi: 10.1371/journal.pone.0082082 (PMC3858271; doi:10.1371/journal.pone.0082082)

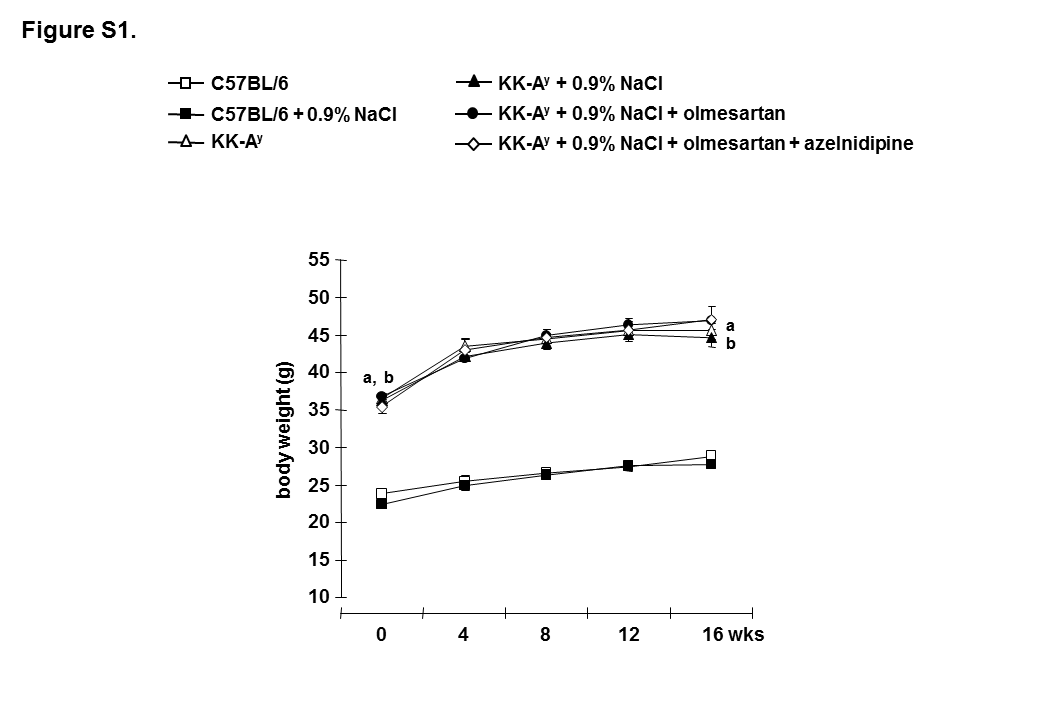

Supplement: Figure S1 — Body weight changes during the experimental period. KK-Ay mice showed higher body weight compared to C57BL mice. However, none of the treatments affected body weight gains in KK-Ay + 0.9% NaCl mice (n=11). a P < 0.05 vs. C57BL/6, b P < 0.05 vs. C57BL/6 + 0.9% NaCl. (TIF) [file pone.0082082.s003.tif]

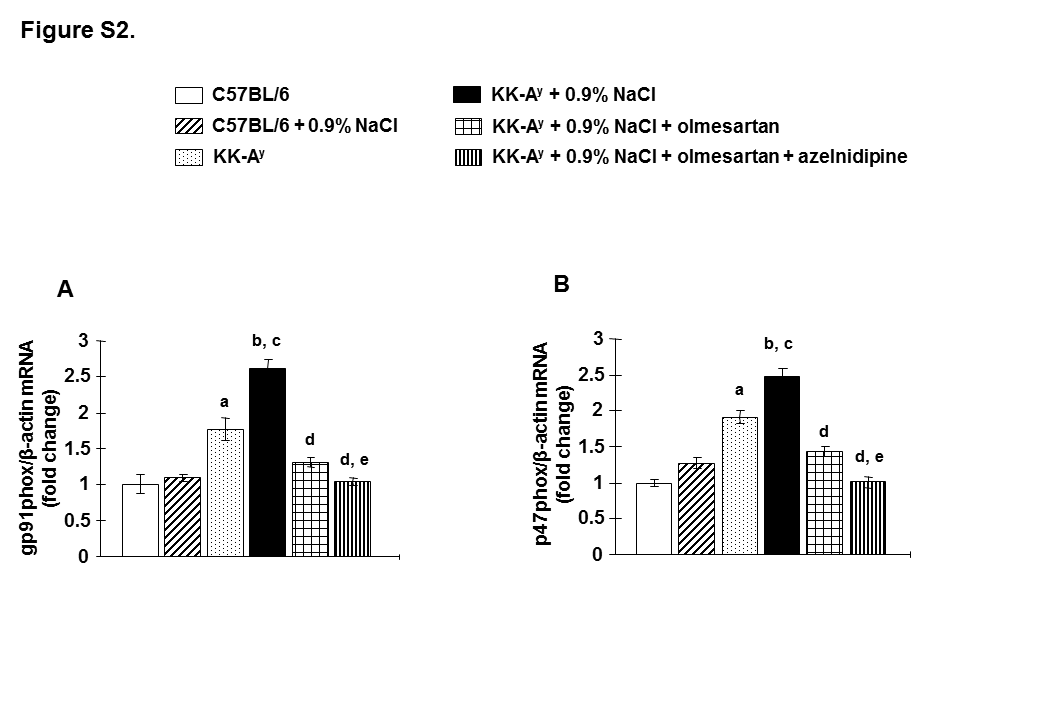

Supplement: Figure S2 — NADPH oxidase subunits gene expression in brain tissues analyzed by RT-PCR. NADPH oxidase subunits gp91phox (A) and p47phox (B) mRNA levels in whole brain tissues. Saline-dinking KK-Ay mice showed upregulation of NADPH oxidase subunit mRNA levels in brain tissues, which were attenuated by treatment with olmesartan. Furthermore, the combination of olmesartan plus azelnidipine completely prevented these changes resulting in levels similar to that in C57BL6 mice (n=8). a P < 0.05 vs. C57BL/6, b P < 0.05 vs. C57BL/6 + 0.9% NaCl, c P < 0.05 vs. KK-Ay, d P < 0.05 vs. KK-Ay + 0.9% NaCl, e P < 0.05 vs. KK-Ay + 0.9% NaCl + olmesartan. (TIF) [file pone.0082082.s004.tif]

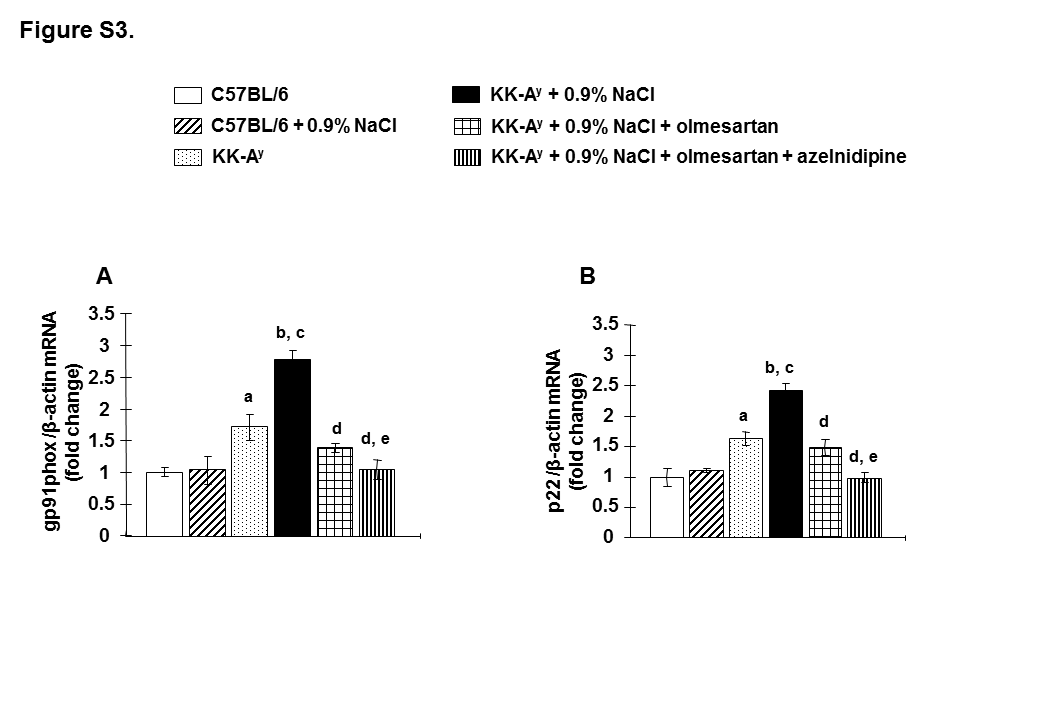

Supplement: Figure S3 — NADPH oxidase subunits gene expression in kidney tissues analyzed by RT-PCR. NADPH oxidase subunits gp91phox (A) and p22phox (B) mRNA levels in renal cortical tissues. In saline-dinking KK-Ay mice, superoxide production in renal tissues was associated with upregulation of NADPH oxidsase subunit genes expression. Treatment with olmesartan markedly attenuated these changes. Furthermore, the combination of olmesartan plus azelnidipine completely prevented these changes resulting in levels similar to that in C57BL6 mice (n=8). a P<0.05 vs. C57BL/6, b P<0.05 vs. C57BL/6 + 0.9% NaCl, c P<0.05 vs. KK-Ay, d P<0.05 vs. KK-Ay + 0.9% NaCl, e P<0.05 vs. KK-Ay + 0.9% NaCl + olmesartan. (TIF) [file pone.0082082.s005.tif]
